# Population Genetic Structure, Abundance, and Health Status of Two Dominant Benthic Species in the Saba Bank National Park, Caribbean Netherlands: *Montastraea cavernosa* and *Xestospongia muta*

**DOI:** 10.1371/journal.pone.0155969

**Published:** 2016-05-25

**Authors:** Didier M. de Bakker, Erik H. W. G. Meesters, Judith D. L. van Bleijswijk, Pieternella C. Luttikhuizen, Hans J. A. J. Breeuwer, Leontine E. Becking

**Affiliations:** 1 Institute for Marine Resources and Ecosystem Studies (IMARES), Wageningen UR, P.O. Box 57, 1780 AB, Den Helder, The Netherlands; 2 Royal Netherlands Institute for Sea Research, P.O. Box 59, 1790 AB, Den Burg, Texel, the Netherlands, and Utrecht University, Utrecht, The Netherlands; 3 Institute for Biodiversity and Ecosystem Dynamics, University of Amsterdam, PO Box 94248, 1090 GE, Amsterdam, The Netherlands; 4 Marine Animal Ecology, Wageningen UR, PO Box 338, 6700 AH, Wageningen, The Netherlands; 5 Naturalis Biodiversity Center, Darwinweg 2, 2300 RA, Leiden, The Netherlands; Biodiversity Research Center, Academia Sinica, TAIWAN

## Abstract

Saba Bank, a submerged atoll in the Caribbean Sea with an area of 2,200 km^2^, has attained international conservation status due to the rich diversity of species that reside on the bank. In order to assess the role of Saba Bank as a potential reservoir of diversity for the surrounding reefs, we examined the population genetic structure, abundance and health status of two prominent benthic species, the coral *Montastraea cavernosa* and the sponge *Xestospongia muta*. Sequence data were collected from 34 colonies of *M*. *cavernosa* (nDNA ITS1-5.8S-ITS2; 892 bp) and 68 *X*. *muta* sponges (mtDNA I3-M11 partition of COI; 544 bp) on Saba Bank and around Saba Island, and compared with published data across the wider Caribbean. Our data indicate that there is genetic connectivity between populations on Saba Bank and the nearby Saba Island as well as multiple locations in the wider Caribbean, ranging in distance from 100s–1000s km. The genetic diversity of Saba Bank populations of *M*. *cavernosa* (π = 0.055) and *X*. *muta* (π = 0.0010) was comparable to those in other regions in the western Atlantic. Densities and health status were determined along 11 transects of 50 m^2^ along the south-eastern rim of Saba Bank. The densities of *M*. *cavernosa* (0.27 ind. m^-2^, 95% CI: 0.12–0.52) were average, while the densities of *X*. *muta* (0.09 ind. m^-2^, 95% CI: 0.02–0.32) were generally higher with respect to other Caribbean locations. No disease or bleaching was present in any of the specimens of the coral *M*. *cavernosa*, however, we did observe partial tissue loss (77.9% of samples) as well as overgrowth (48.1%), predominantly by cyanobacteria. In contrast, the majority of observed *X*. *muta* (83.5%) showed signs of presumed bleaching. The combined results of apparent gene flow among populations on Saba Bank and surrounding reefs, the high abundance and unique genetic diversity, indicate that Saba Bank could function as an important buffer for the region. Either as a natural source of larvae to replenish genetic diversity or as a storehouse of diversity that can be utilized if needed for restoration practices.

## Introduction

The Saba Bank, Caribbean Netherlands, is a large (2,200 km^2^, along the 100 m isobath) submerged carbonate platform [1, 2] and harbors a rich diversity of marine species [3–6]. In 2010, the Dutch Government declared the Bank a Protected Area and it has been registered as such in the Specially Protected Areas and Wildlife (SPAW) protocol of the Cartagena Convention for the Protection and Development of the Marine Environment of the Wider Caribbean (http://www.spaw-palisting.org/area_public/show/id/10). Saba Bank received the designation of a Particular Sensitive Sea Area (PSSA) at the International Maritime Organization (IMO) in 2012 and was acknowledged as an Ecological or Biological Significant Area (EBSA) at the Convention on Biological Diversity (CBD). The bank also received special attention within the management plan of the Caribbean Netherlands to ensure the protection of its unique biodiversity [7–9]. In addition, the Dutch Ministry of Economic Affairs, Agriculture and Innovation has instigated “The Saba Bank Research Program 2011–2016” in order to determine the health status of Saba Bank, to, among other objectives, gain insight in key ecological processes.

Due to its offshore position—5 km from Saba Island—and upper-mesophotic reef system (20–50 m deep), Saba Bank coral reefs appear to have suffered relatively little anthropogenic disturbance compared to the fringing reefs of the surrounding islands. This is reflected, for instance, by the relative absence of diseases [4, 5, 9], as well as the presence of large predators (*e*.*g*. sharks, groupers and snappers) [8, 10], suggesting Saba Bank could fulfill an essential role in the resilience of nearby reefs as a source of larvae and genetic diversity. Coral reef organisms are strongly dependent on recruitment from surrounding reefs after local disturbances (*e*.*g*. hurricanes) [11]. Understanding patterns of connectivity is therefore essential to implement effective reef conservation strategies [12]. If Saba Bank is to serve as a reservoir of diversity for the surrounding reefs, it is important to understand how populations on the bank are positioned in the genetic structure of the wider Caribbean populations and how stress and diseases are currently affecting the populations. The aim of the current study was to examine the genetic connectivity, density, and health status (*i*.*e*. presence of diseases or traces of recent bleaching) of populations of two prominent benthic reef species on Saba Bank; the star coral, *Montastraea cavernosa* (Linnaeus 1767), and the giant barrel sponge, *Xestospongia muta* (Schmidt 1870). Both species have been recorded on Saba Bank in surveys since the 1970s [3, 5, 9].

*M*. *cavernosa* is a common reef-building scleractinian coral in the tropical and sub-tropical Atlantic [13] and can account for up to 95% of the total coral cover in some regions (*e*.*g*. eastern Brazil) [14]. In the Florida Keys and the Cayman Islands benthic surveys indicated colony densities between 0.14–6.32 m^-2^ [15–17]. *M*. *cavernosa* is a broadcast spawner, releasing sperm and eggs into the water column where fertilization and development take place [18]. Planktonic planulae of broadcast spawning corals can survive up to 100 days before final settlement, allowing for potential dispersal over considerably large distances (> 600 km) [19]. Although *M*. *cavernosa* is ubiquitous throughout the Atlantic and a clear genetic structure exists between the Caribbean, Brazilian and eastern Atlantic regions—separated by 4 000–10 000 km—indicating that gene flow is restricted at the larger scale [20]. On a lower scale, however, high levels of genetic connectivity have been found amongst the majority of populations within the Caribbean [21, 22], with the exception of some locations (*e*.*g*. Barbados or the Little Cayman Islands) which appear to rely more on self-recruitment [21, 23].

*M*. *cavernosa* is vulnerable to Black Band Disease and White Plague Disease throughout the Cairbbean, affecting up to 2.9% and 1.8% of the colonies respectively on reefs of Jamaica [24, 25]. Ferreira and colleagues [26] also describe the presence of a Dark Spot and Yellow Band Disease affecting several colonies in the Brazilian Fernando de Noronha archipelago. Additionally, *M*. *cavernosa* colonies are susceptible to bleaching [27–29], although these seem to have a higher tolerance to increased temperatures compared to other corals (*e*.*g*. *Orbicella annularis* or *Agaricia spp*.) [30]. There might be some degree of overestimation in previous surveys, as natural whitish coloration might have been wrongly ascribed to bleaching [31].

In addition to corals, sponges play a crucial role in coral reef ecosystems [32–37]. *X*. *muta* is one of the largest and most common members on Caribbean reefs and is often referred to as the ‘redwood of the reef’ due to its long lifespan [38–40]. It contributes significantly to the habitat complexity and can overturn substantial volumes of reef water (up to 0.078 L s^−1^ L^−1^ sponge tissue) [41, 42], hence playing a crucial role in the reef system. *X*. *muta* has been recorded to reach densities as high as 0.28 ind. m^-2^ [43]. Although there is little data on the larval survival of *X*. *muta*, the larval dispersal is expected to be limited, similar to the congener *Xestospongia testudinaria* [44, 45]. To date, Lopez-Legentil. (2009) [46] published the only study on genetic connectivity of *X*. *muta* populations in the western Atlantic. They report strong genetic structure among the majority of the sampled populations, using the I3-M11 partition of COI.

The peripheral tissue of *X*. *muta* harbors cyanobacteria of the genus *Synechococcus* that gives these barrel sponges the characteristic reddish brown coloration [47]. Similar to corals, *X*. *muta* is known to expel part of its symbiont community during region wide bleaching events [48–51]. Within the Caribbean a number of massive bleaching events have already been reported, as reviewed by Angermeier *et al*. (2011) [52]. Cowart *et al*. (2006) [49] described two types of bleaching in barrel sponges on Conch Reef, Florida Keys: cyclic bleaching, which seems to be temporary (affecting ± 25% of the population) and fatal bleaching (affecting < 1% of the population), which is synonymous with Sponge Orange Band Disease (SOB) and usually results in sponge mortality [49, 50, 53].

In the present study, we aimed to assess the role of Saba Bank in recruitment of two common benthic species (*M*. *cavernosa* and *X*. *muta*). With the aid of molecular techniques and photographed transects, we quantified 1) the level of genetic diversity within the populations on Saba Bank; 2) the degree of genetic connectivity between populations on Saba Bank and surrounding reefs, based on novel sequences and published sequences of populations across the wider Caribbean; 3) the current density and health status of the populations of *M*. *cavernosa* and *X*. *muta* on Saba Bank.

## Materials and Methods

### Ethics statement

This research is part of the BO (Beleidsondersteunend Onderzoek) program Caribbean Netherlands of the Ministry of Economic Affairs (EZ) under project number BO-11-011.05–033. LEB was supported by the Veni-grant of the Netherlands Organization for Scientific Research (#863.14.020). Research and tissue collection in the waters of Saba Bank was carried out under approval of the Ministry of Economic Affairs, National Office for the Caribbean Netherlands on August 30th 2013 on the basis of artikel 30 lid 1 Wet Maritiem Beheer BES registered under reference no. RWS-2013/42681. All sampled animals are invertebrates. Tissue collection was kept at a minimum and will not have permanent negative consequences to any of the sampled colonies or individuals.

### Sample collection and handling

**Fig 1:**
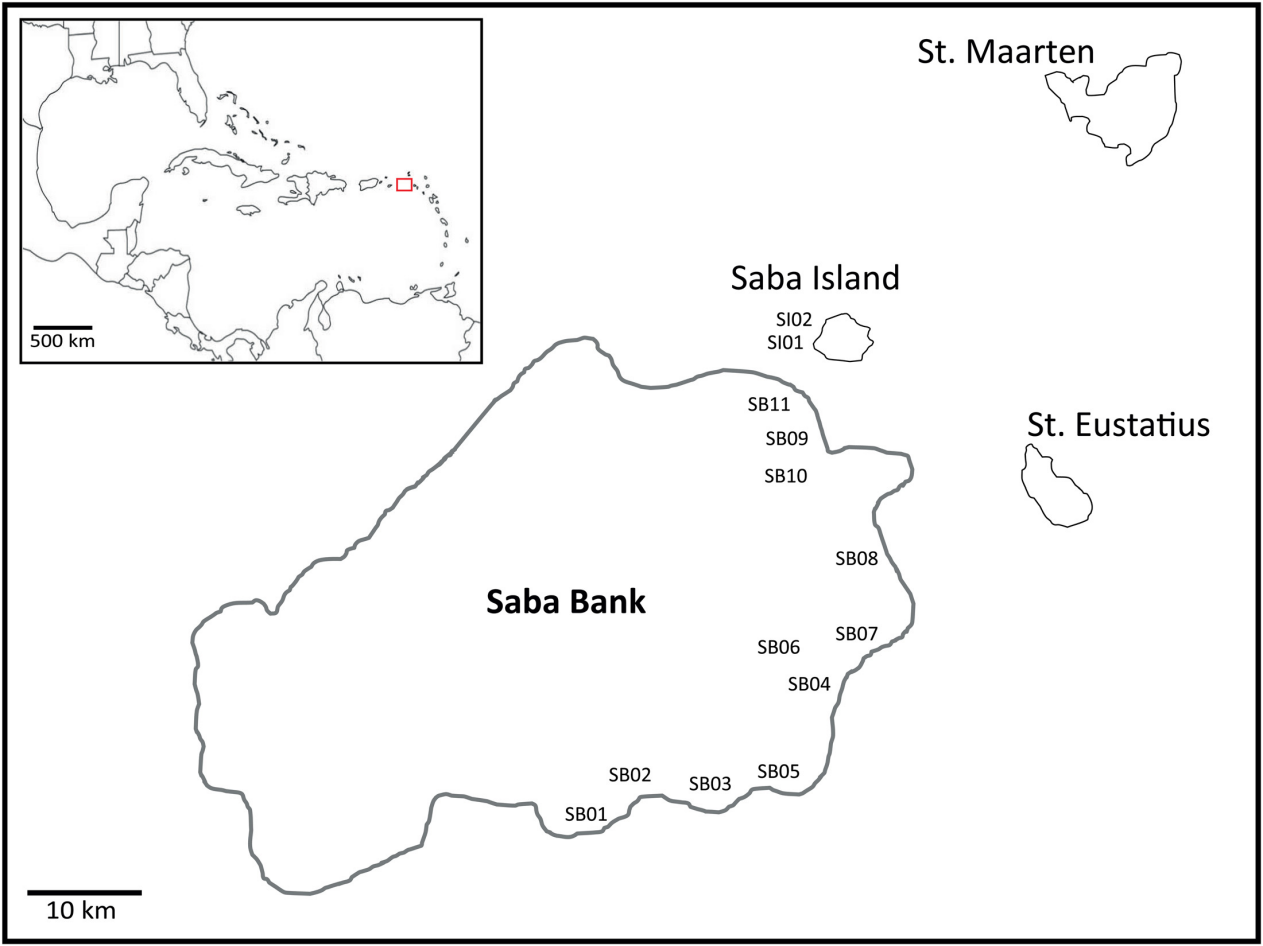
Sample locations on Saba Bank (SB01-SB11) and around Saba Island (SI01 and SI02). Included is a scheme of Saba Bank’s position with respect to nearby islands and in the wider Caribbean region (indicated by red square in overview).

From 19–27 October 2013, IMARES (Wageningen UR), organized a research expedition to the Saba Bank (17° 25’ N, 63° 30’ W) to investigate the ecological functioning of the Bank, on board of the “Caribbean Explorer II”. The expedition is a follow-up of a survey of the Bank in 2011 and is part of “The Saba Bank Research Program 2011–2016” initiated by the Dutch Ministry of Economic Affairs (EZ). Saba Bank is located approximately 5 km south-west of Saba Island (Fig 1). It is the largest submerged carbonate platform in the Atlantic Ocean [2, 7]. The majority of the bank is occupied by algal fields and sand dominated patches, except for the 55 km long coral ridge on the eastern and southern edge [1, 8]. We conducted surveys at 11 sites (SB01 –SB11) on Saba Bank (Fig 1) of which the location was determined during the previous expedition, ‘Saba Bank I expedition’ in October 2011, based on depth (15–30 m) and benthic cover of corals, sponges and macro algae. These sites are considered to properly represent the habitat variation found on Saba Bank reef crest. At each site, all *X*. *muta* and *M*. *cavernosa* colonies within a 50 m belt transect were photographed to obtain local densities and quantify disease or bleaching incidences. Tissue of corals (*M*. *cavernosa)* and sponges (*X*. *muta)* for molecular analysis was collected haphazardly at each site, but within the vicinity of the belt transect. In addition, molecular samples were collected at two sites near Saba Island. On the Saba Island sites no transects were laid out and data on densities or disease is absent. To minimize the chance of sampling clones, a minimum distance of 10 m was kept between colonies. All collected samples were id-labelled (including location, depth and coloration) and source colonies were extensively photographed–*in situ* and after collection—to allow for comprehensive identification and disease recognition. Several polyps of the coral colonies’ edge were collected using a hammer and chisel. Sponge tissue was obtained using an apple corer in order to get symbiont-rich surface as well as internal tissue [54]. To minimize the chance of sampling clones, minimum distance between colonies was kept at 10 m. For DNA analysis, a 0.5 cm^3^ piece of each sample was preserved in 2 mL reaction tubes with RNA*later*^tm^ (QIAGEN). The remaining tissue was kept on 96% ethanol for morphological identification. All samples were kept at 4°C directly after collection and during transport, subsequently they were stored at -20°C.

### DNA extractions and PCR amplification

Total DNA was extracted using the GenElute Mammalian Genomic DNA Miniprep kit (Sigma) following the manufacturer’s protocol, with an additional step of grinding the tissue gently with a sterile plastic pestle to improve homogenization after the addition of Lysis ‘T’ solution. The solution was incubated for approximately 4 hours at 55°C, or until complete cell lysis. All laboratory work was performed at the *Royal Netherlands Institute for Sea Research* (NIOZ) Texel, the Netherlands.

The 892 bp (base pairs) long internal transcribed spacer 1–5.8S ribosomal RNA—internal transcribed spacer 2 (ITS hereafter) was amplified for *M*. *cavernosa* using the primers *ITSant1S* (5’-GGT ACC CTT TGT ACA CAC CGA CCG TCG CT-3’) and *ITSant2S* (5’-GCT TTG GGC GGC AGT CCC AAG CAA CCC GAC TC-3’) [21, 55]. The 50 μL PCR reaction volume contained 5.0 μL 10x buffer, 5 μL dNTP (2.5 mM), 0.25 μL (50 μM) of each Primer, 0.25 μL Biotherm+ Taq, 1.0 μL BSA and 2 μL of DNA template (undiluted). The PCR profile had an initial denaturation step of 3 min. at 94°C, followed by 36 cycles of 94°C (30s), 50°C (30s) and 72°C (45s) and a final extension of 5 min. at 72°C. The 544 bp long I3-M11 partition of the mitochondrial cytochrome oxidase I (COI) gene of *X*. *muta* was amplified using the universal metazoan primer *C1-J2165* (5’-GAA GTT TAT ATT TTA ATT TTA CCD GG-3’) [56] and the reverse primer *C1-Npor2760* (5’-TCT AGG TAA TCC AGC TAA ACC-3’) [57]. 50 μL PCR reaction volume contained 5.0 μL 10x PCR buffer, 5.0 μL dNTP (2.5 mM), 0.5 μL (50 μM) of each Primer, 0.25 μL BiothermPlus Taq, 2.0 μL of DNA template. The thermal cycler profile consisted of an initial denaturation step of 3 min at 95°C followed by 35 cycles of 95°C (30s), 42°C (30s) and 68°C (90s) and a final extension at 72°C (10 min). All PCR reactions were carried out in T-Gradient Thermo-block (Biometra) or Doppio Fuse 8.0A thermal cyclers. PCR products were sequenced forward and reverse by BaseClear B.V. Leiden, The Netherlands.

### Sequence preparation

Consensus sequences were constructed from the forward and reverse sequences, using the software programs *Auto-assembler DNA Sequence Assembler* ver. 2.1 (Applied Bio-systems, Perkin-Elmer) and *Chromas Pro* ver. 1.7.5 (Technelysium Pty. Ltd, Tewantin, Queensland, Australia). Final consensus sequences were aligned (93% similarity 5.0/-9.026186) in Geneious^®^ ver. 7.0.6 (Biomatters) with homologous sequences obtained from GenBank^®^ (http://www.ncbi.nlm.nih.gov/genbank/). In order to recover all previously published sequences of ITS and COI of *M*. *cavernosa* and *X*. *muta*, respectively, a search was conducted with the specific genetic marker and species as search terms in GenBank. Ambiguities in consensus sequences were, where possible, visually resolved using the original chromatogram files. When the lower peak was over 80% in height of the higher peak at an ambiguous site, and no more than a single ambiguous site was found in that particular sequence the alleles were separated. If multiple nucleotide ambiguities in one sequence could not be resolved or no consensus could be built, these sequences were excluded from subsequent analyses (one I3-M11 sequence and five ITS sequences). The low number of ambiguities found in the ITS data is in correspondence with the observations of Goodbody-Gringley and colleagues (2012) [21] who found, for *M*. *cavernosa*, a maximum of one ambiguous peak within any individual ITS sequence. Novel sequences are available in GenBank under Accession Numbers KT254598-KT254638 and KT271771-KT271838 (S1 Table).

### Genetic variation and population structure

Genetic diversity on Saba Bank and populations in the wider Caribbean region was determined based on estimates of haplotype diversity (*h*) [58] and nucleotide diversity (*π*) [58] using the software Arlequin ver. 3.5.1.2 [59]. Analysis of molecular variance (AMOVA) [60] was conducted among all 11 Saba Bank sample sites to determine presence of genetic population structure among the sample sites. Comparisons among all sampled locations were tested based on pairwise Φ_ST_ statistics (10 000 bootstrap permutations). All AMOVA, exact and Φ_ST_ statistics tests were also conducted in Arlequin. Maximum likelihood trees were constructed in MEGA ver. 6.06 [61] and subsequently used to construct haplotype networks in HaplotypeViewer [62]. The most suitable model (JC+G for ITS and K2+G+I for I3-M11) was selected in jModelTest ver. 2.1.2 [63], based on the Akaike Information Criterion (AIC).

### Migration analyses

In order to examine the possibility of asymmetrical migration, we carried out an isolation-with-migration analyses with the model IMa2 [64]. Doing so also allowed us to obtain rough estimates of the time scale of colonization of the study area as well as effective population sizes (N_e_). To convert model parameters into demographic units, an inheritance scalar of 0.5 for mitochondrial DNA in hermaphroditic species (i.e., θ = 2N_e_μ)) and a generation time of 1 year were assumed. Substitution rate for COI in *X*. *muta* was estimated at 0.0194% per million years (MY), based on a maximum interspecific pairwise distance of 21% in the Porifera [65] and the oldest sponge fossil dating to 540 million years ago (MYA) [66]; for IMa2, that translates to a per-locus mutation rate of 0.11*10^−6^ per year per 544 bp. For *M*. *cavernosa*, the results of the migration analysis were highly inconsistent and therefore, the applied methods and results for ITS will not be discussed here.

Only pairs of populations were compared in order to reduce the number of parameters in the models as much as possible. The selection of locations was made on the basis of a sample size of n > 20 and their geographic position with respect to Saba Bank. For *X*. *muta*, Saba Bank was compared to Stirrup Cay and the pooled populations of San Salvador and Plana Cay (hereafter referred to as SSPC). Analyses were done using four independent runs for each population pair, each run consisting of ten Markov Chain Monte Carlo chains with geometric heating (h1 = 0.99, h2 = 0.75) of two million steps after an initial burn-in period of five million steps. The infinite sites model of substitution was used. Convergence of parameter distributions was ensured by: examining effective sample size (ESS) values, autocorrelation values and chain swapping, checking trend line plots for absence of trends and by comparing parameter estimates generated from the genealogies produced during the first and second half of runs.

### Population density and health status

At each Saba Bank station one transect line of 50 m was placed on the reef surface in order to determine the densities of *M*. *cavernosa* colonies and *X*. *muta*. Every meter a high resolution photograph was taken (S1 Fig) from which densities were extracted visually. Only sponges and corals present in a 1 m^2^ section in the center of each image were counted. Every square was aligned with each meter of transect to prevent repeated counting. *M*. *cavernosa* colonies smaller than 4 cm were considered juvenile [67]. No transects were recorded on Saba Island, hence, data on densities around Saba Island is absent.

All sampled colonies of *M*. *cavernosa* and individuals of *X*. *muta* as well as those on the transect pictures (covering 50 m^2^ per site) were analyzed for signs of disease or bleaching. Previous mortality was identified by the loss of tissue where polyps were still recognizable. Partial overgrowth of the tissue by other benthic components (*e*.*g*. algae or cyanobacteria) was also recorded. Individual *X*. *muta* were examined for both types of assumed bleaching as described by Cowart *et al*. (2006) [49] and McMurray *et al*. (2011) [51]. Cyclic bleaching can be recognized by the circle shaped spots with loss of the typical brownish-red coloration in parts of the sponge tissue and Sponge Orange Band Disease by a clear orange band separating completely bleached (dead) from still untouched sponge tissue.

## Results

### Genetic diversity

**Fig 2:**
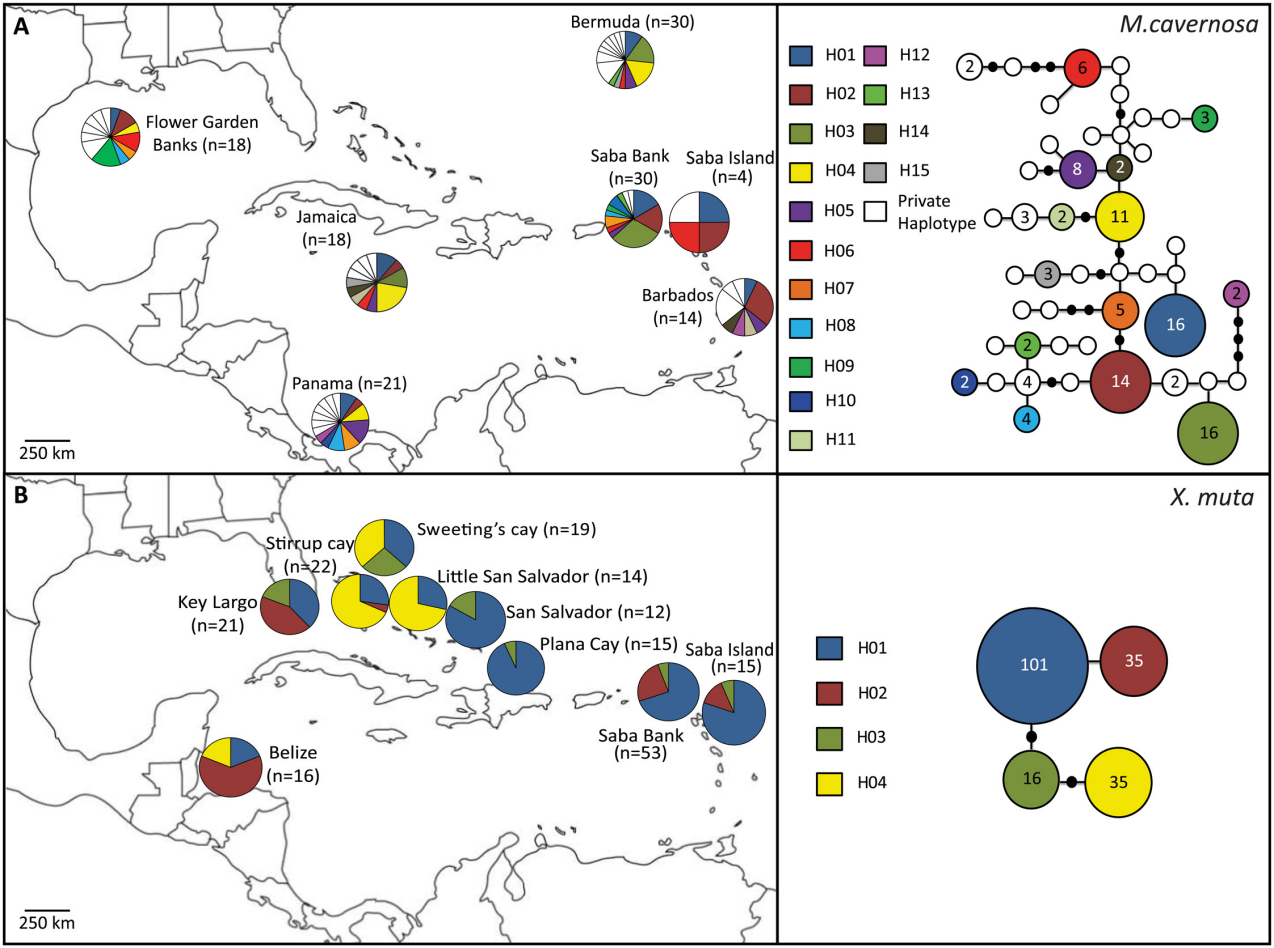
Frequency and distribution of haplotypes in populations of *Montastraea cavernosa* (A) and *Xestospongia muta* (B) in the wider Caribbean region. Haplotype frequencies provided as pie-chart per location, number of samples in brackets. Haplotype network of ITS (A) and I3-M11 (B), baes on all sequences collected throughout the wider Caribbean region. Size of circle reflects the number of individuals with a specific haplotype. Each line represents the genetic distance between haplotypes. Specific haplotype colors match those presented in the pie-charts. White circles (ITS) are haplotypes private to a certain location.

For *M*. *cavernosa* 34 sequences of 832 bp fragment length (ITS), representing 13 haplotypes, were obtained from Saba Bank and Saba island (Table 1, Fig 2A). The combined data set, including previously published sequences from the wider Caribbean [20, 21], yielded a total of 46 haplotypes (Fig 2A) with 26 polymorphic sites. Haplotypes H01-H03 (GenBank access#: HM447268, HM447255 and HM447299) were dominant on Saba Bank and throughout the western Atlantic (Fig 2A). Two unique haplotypes were present on Saba Bank (KT254613, KT254632) and one on Saba Island (KT254636).

**Table 1:** Number of obtained sequences, number of haplotypes and genetic diversity indices for *Montastraea cavernosa* (ITS) and *Xestospongia muta* (I3-M11).

Location	*N*	*n*	*h*	*π*
*M*. *cavernosa*				
Saba Bank^1^	30	12(2)	0.8828	0.0055
Saba Island^1^	4	4(1)	1.0000	0.0069
Barbados^2^	14	9(3)	0.9011	0.0051
Bermuda^2^	30	15(9)	0.9287	0.0062
Flower Garden Banks^2^	18	13(7)	0.9608	0.0059
Jamaica^2^	18	13(4)	0.9542	0.0055
Panama^2^	21	15(8)	0.9667	0.0056
*X*. *muta*				
Saba Bank^1^	53	3	0.4579	0.0010
Saba Island ^1^	15	3	0.3619	0.0010
Key largo^3^	21	3	0.6667	0.0021
Belize^3^	16	3	0.5750	0.0033
Sweetings cay^3^	19	3	0.6959	0.0036
Plana cay^3^	15	2	0.1333	0.0005
San Salvador^3^	12	2	0.3030	0.0011
Little San salvador^3^	14	2	0.4396	0.0032
Stirrup cay^3^	22	3	0.4805	0.0036

*N*, number of obtained sequences; *n*, number of haplotypes (private haplotypes given in parentheses); *h*, haplotype diversity, *π*: nucleotide diversity;

^1^This study;

^2^Goodbody-Gringley *et al*. (2012);

^3^Lopez-Legentil and Pawlik (2009);

^4^Montalvo and Hill (2011).

For accession numbers see S1 Table.

For *X*. *muta* a total of 68 (Saba Bank and Saba Island) sequences of 544 bp (COI) were obtained, representing 3 haplotypes from Saba Bank and Saba Island (Table 1, Fig 2B). No unique haplotypes were discovered. The combined data set including previously published sequences from the wider Caribbean [44, 46] yielded a total of 4 haplotypes (Fig 2B) with 5 polymorphic sites. Three haplotypes (H01, H02; GenBank access#: EU716652, EU716653) were dominant on Saba Bank and also in the wider western Atlantic (Fig 2B).

### Population structure

For both *M*. *cavernosa* (ITS) and *X*. *muta* (COI) there was no genetic structure on Saba Bank. AMOVA tests among sample the 11 locations on the Saba Bank showed that genetic variation was almost exclusively explained within sites (S2 Table) for both *M*. *cavernosa* (92.9%) and *X*. *muta* (100%). Subsequent pairwise comparisons among the Saba Bank sites resulted in non-significant Φ_ST_ values (S3 Table). The pairwise Φ_ST_ was only significantly different from zero for *X*. *muta* among sites SB07 and SB08, most likely due to the small sample size of SB08 (n = 2). Since no significant genetic structure was found for either of the species we could considered all 11 sites as one Saba Bank population in the subsequent analyses. An AMOVA among the Saba Bank population and populations from locations in the wider Caribbean showed that the vast majority (97.9%) of variation exists within locations of *M*. *cavernosa* rather than among (2.1%), supporting previous findings of absence of strong genetic structure in the greater Caribbean. For *X*. *muta* variation among populations was much higher (39%), indicative for presence of genetic structure between locations (for an overview of AMOVA results see S4 Table). There was, however, no significant difference among the populations of Saba Bank and the neighboring Saba Island, neither for *M*. *cavernosa* nor *X*. *muta* (Tables 2 and 3). For *M*. *cavernosa* significant albeit low Φ_ST_ values were obtained when comparing the population of Saba Bank to the populations in Barbados, Flower Gardens Bank (Gulf of Mexico), and stronger differentiation between Saba Bank and Panama (Table 2). The population of *X*. *muta* on Saba Bank showed no significant differentiation from Florida, yet it did show strong and significant differentiation with Belize and Bahamas (Table 3).

**Table 2:** Matrix of pairwise population differentiation values (Φ_st_) between populations of *Montastraea cavernosa* at Saba Bank and in the wider Caribbean and Gulf of Mexico. Significant values (p < 0.05) are provided in bold.

	Saba Bank	Saba Island	Barbados	Bermuda	Gulf of Mexico	Jamaica	Panama
*ITS*							
Saba Bank	*-*						
Saba Island	0.0032	*-*					
Barbados	**0.0570**	-0.0644	-				
Bermuda	0.0238	-0.0602	0.0103	-			
FGB	**0.0544**	-0.0672	0.0226	0.0099	-		
Jamaica	0.0340	-0.0644	-0.0072	-0.0170	0.0137	-	
Panama	**0.0973**	-0.0742	-0.0115	0.0066	0.0105	0.0156	-

**Table 3:** Matrix of pairwise population differentiation values (Φ_st_) between populations of *Xestospongia muta* at Saba Bank and in the wider Caribbean and Gulf of Mexico. Significant values (p < 0.05) are provided in bold.

	Saba Bank	Saba Island	Florida	Belize	Bahamas (Sweetings Cay)	Bahamas (Plana Cay)	Bahamas(San Salvador)	Bahamas (L. San Salvador)	Bahamas (Stirrup Cay)
*I3-M11*									
Saba B.	-								
Saba I.	-0.0212	-							
Florida	0.0557	0.0693	-						
Belize	**0.1981**	**0.1835**	0.0204	-					
Bahamas SC	**0.5031**	**0.3893**	**0.3321**	**0.2818**	-				
Bahamas PC	0.0673	-0.0216	**0.1756**	**0.2847**	**0.4065**	-			
Bahamas SS	0.0828	-0.0069	0.1120	**0.2210**	**0.2946**	-0.0277	-		
Bahamas LSS	**0.6975**	**0.6179**	**0.5445**	**0.4369**	0.0677	**0.6452**	**0.5535**	*-*	
Bahamas STC	**0.6387**	**0.5450**	**0.4906**	**0.3942**	0.0532	**0.5638**	**0.4836**	-0.0583	-

### Patterns of migration

The pairwise comparison between *X*. *muta* from Saba Bank and Stirrup Cay (Bahamas) gave results that were highly reproducible between runs. The coalescent model results suggest that the two populations presently inhabiting Saba Bank and Stirrup Cay were established 1.7–2.0 MYA, but that since this time they continue to be connected through low levels of gene flow. The present-day population size (N_e_) is estimated to be 0.74 to 0.77 million for Stirrup Cay and 1.5 to 1.6 million for Saba Bank (S5 Table). The simulations suggest that gene flow from Stirrup Cay to Saba Bank is virtually non-existent, while gene flow in the opposite direction does occur, but in low numbers. Simulations for the pairwise comparison between *X*. *muta* from Saba Bank and SSPC (San Salvador and Plana Cay samples from the Bahamas combined) gave inconsistent results and broad posterior probability intervals (S2 Fig and S5 Table). The results suggest gene flow from SSPC into Saba Bank, while the simulation results are inconclusive about gene flow in the opposite direction.

### Population density and disease on Saba Bank

**Fig 3:**
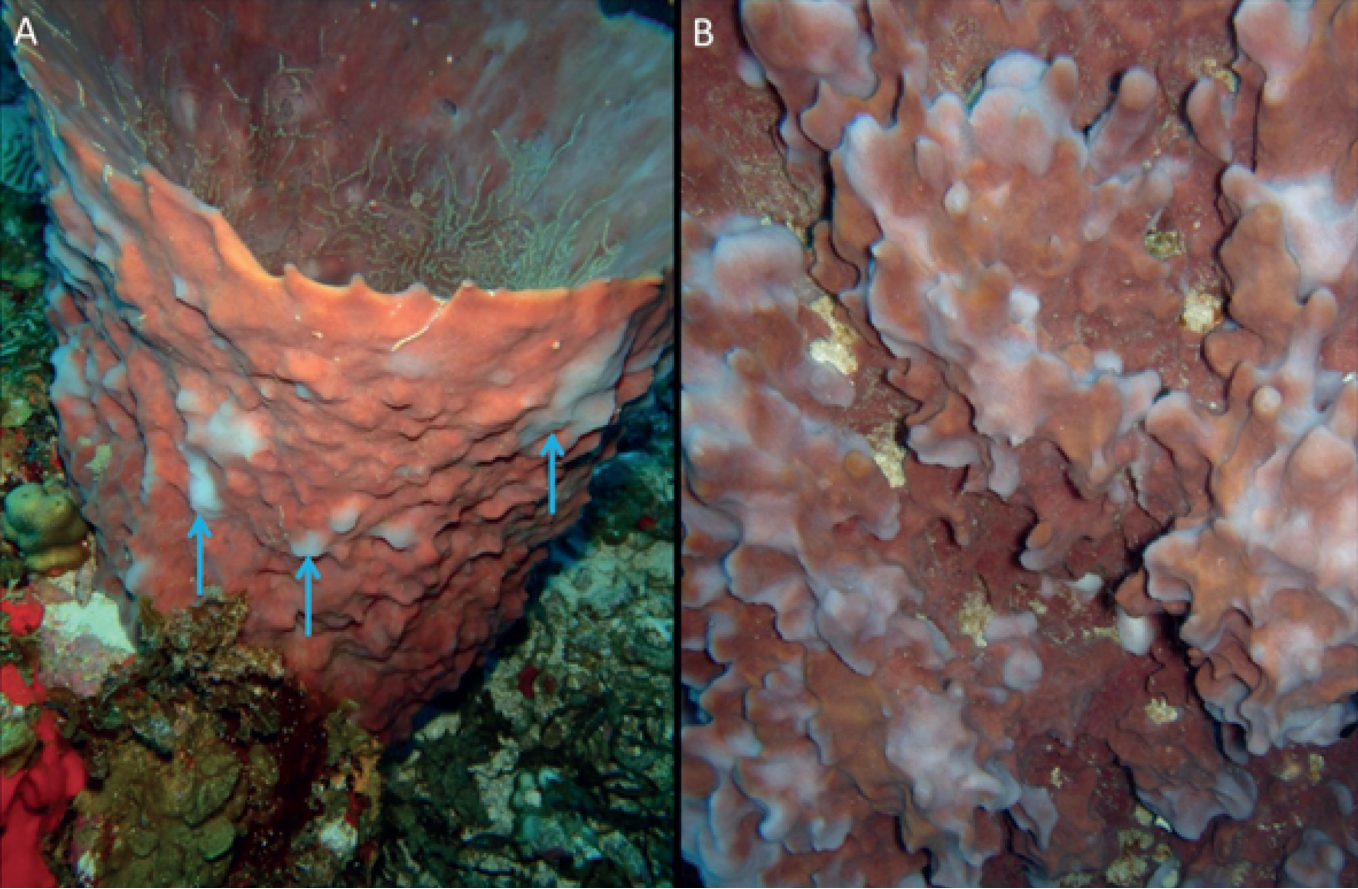
Bleaching in Xestospongia muta. (A) Cyclic bleaching (blue arrows). (B) Close up of cyclic bleaching close up.

Based on our survey of 11 sites, of 50 m^2^ transects at each site (total area surveyed 550 m^2^), the mean density on Saba Bank reef crest of *M*. *cavernosa* was 0.27 [95% CI: 0.12–0.52] ind. m^-2^ and of *X*. *muta* 0.09 [95% CI: 0.02–0.32] ind. m^-2^ (Table 4). A total number of 261 *M*. *cavernosa* and 186 *X*. *muta* were photographed and assessed for bleaching, disease, and overgrowth. Juvenile *M*. *cavernosa* (< 4 cm) accounted for 4.5% of all analyzed colonies. No disease was observed in any of the *M*. *cavernosa* colonies. Previous tissue loss (78% of colonies) and current overgrowth of a part of the colony (48.1% of colonies) was frequently observed in *M*. *cavernosa*. Cyanobacteria accounted for the vast majority of overgrowth (56.7%) followed by fleshy macro algae or turf algae (23.6%), sponges (15.8%), gorgonians (2.4%) and crustose coralline algae (1.6%). *X*. *muta* showed extensive presence of presumably cyclic bleaching [49], which fit the description of ‘spottily bleached’ defined by McMurray *et al*. [51] as ‘numerous localized patches or spots of white tissue’. Approximately all of the sampled sponges (92%) and the majority of sponges visible on the transect pictures (75%), showed ‘spottily bleached’ tissue (Table 4, Fig 3). Orange band Disease was encountered on three sponges, all at Saba Bank site SB06, of which two had suffered complete mortality. Many smaller (< 10 cm) *X*. *muta* (> 20% on the transect pictures) showed partial tissue loss associated with overgrowth by algae or other sponges.

**Table 4:** Main characteristics of *Montastraea cavernosa* and *Xestospongia muta* population at the 11 Saba Bank sites. All values for densities and signs of stress are based on image analysis of 50 m^2^ transect surveys at each site and do not correspond to the samples collected for genetic analyses. (n) number of *M*. *cavernosa* and *X*. *muta*; densities per square meter; percentage of *M*. *cavernosa* colonies with tissue loss and overgrowth; and percentage of *X*. *muta* with cyclic bleaching or Sponge Orange Band Disease (SOB [49]). No disease was detected in any of the recorded *M*. *cavernosa* colonies.

			*M*. *cavernosa*				*X*. *muta*			
Site	Latitude	Longitude	n *M*.*cavernosa*	Density (n m^-2^)	Tissue loss (%)	Overgrowth (%)	n *X*.*muta*	n m^-2^	Cyclic Bleaching (%)	SOB (%)
SB1	17°23'48''N	63°44'61''W	46	0.92	80	54	7	0.14	57	0
SB2	17°26'83''N	63°40'87''W	48	0.96	56	27	10	0.2	70	0
SB3	17°26'19''N	63°34'42''W	8	0.16	88	63	5	0.1	60	0
SB4	17°34'58''N	63°25'11''W	9	0.18	100	78	0	0	0	0
SB5	17°27'11''N	63°28'08''W	23	0.48	87	61	23	0.46	52	0
SB6	17°38'41''N	63°28'98''W	1	0.02	100	0	18	0.36	22	17
SB7	17°39'69''N	63°19'63''W	28	0.56	89	48	36	0.72	67	0
SB8	17°45'83''N	63°22'22''W	3	0.06	100	67	0	0	0	0
SB9	17°52'55''N	63°27'02''W	11	0.22	91	73	5	0.1	80	0
SB10	17°50'58''N	63°25'38''W	20	0.40	80	55	3	0.06	67	0
SB11	17°55'94''N	63°28'63''W	10	0.16	70	50	11	0.18	91	0

## Discussion

### Genetic diversity and connectivity

This is the first study to address the population genetic structure among populations of Saba Bank and the surrounding region. We examined the role of Saba Bank as a buffer for diversity in the Caribbean, via population genetic analyses, migration analyses and surveys on abundance and health of two prominent benthic species. The populations of both the coral *Montastraea cavernosa* and the sponge *Xestospongia muta* appear to be connected along the whole eastern and southern rim of the Saba Bank, as well as among the populations on Saba Bank and the nearby island of Saba. Our results suggest that Saba Bank harbors viable populations that could function as a source of diversity, as the genetic diversity of the populations of *M*. *cavernosa* (*π* = 0.055, *h* = 0.883) and *X*. *muta* (*π* = 0.001, *h* = 0.362–0.458) on Saba Bank are comparable to the ranges of diversity found for these species in other Caribbean locations (*M*. *cavernosa*: *π* = 0.005–0.006, *h* = 0.901–0.967 [21]; *X*. *muta*: *π* = 0.0005–0.004, *h* = 0.133–0.696 [46]). Furthermore, migration analyses of *X*. *muta* suggested gene flow to occur from Saba Bank to the Bahamas.

For *M*. *cavernosa* there appears to be unrestricted gene flow among the locations of Saba and the majority of the studied locations in the wider Caribbean. The putatively high level of genetic exchange within the studied region—despite geographical separation of over 2000 km—could be the result of specific life history traits in combination with ocean currents. Being a broadcasting species [18], the larvae of *M*. *cavernosa* have the ability to drift with currents over distances of several hundreds of kilometers [13, 20]. Hydrological features, such as direction of major currents (SE-NW in the Caribbean region [68]) can, however, cause limitations to dispersal and thus form barriers to gene flow. Cowen *et al*. (2006) [69] suggest recruitment limitations due to the partial hydrological isolation of the Leeward Islands—including Saba Bank—from the wider Caribbean which could result in considerable levels of self-recruitment. Such restrictions might explain the significant, albeit weak, differentiation we found among Saba Bank populations and the populations from Barbados, the Gulf of Mexico (Flower Gardens Bank) and, slightly stronger, Panama. Different degrees of genetic isolation of *M*. *cavernosa* populations within the Caribbean region have also been recorded by Goodbody-Gringley *et al*. (2011) [21], Brazeau *et al*. (2014) [23] and Serrano *et al*. (2014) [22] probably due to local hydrology, limiting larval dispersal away and, at certain locations, stronger reliance on self-recruitment as well [21, 69].

For *X*. *muta*, connectivity seems to be limited between the populations on Saba Bank and Belize, as well as most of the locations in the Bahamas. The migration analyses suggest that colonization of the Saba Bank was not recent, with population subdivision time for *X*. *muta* from Saba Bank versus Stirrup Cay estimated at 1.7–2.0 million years ago. Note that the absolute numbers inferred in the present study depend heavily on the assumption that the generation time is one year. Unfortunately, not much is known about generation time of sponges in natural habitats. Additionally, the inferences rely on a single locus only and may thus depend on specifics of that locus. Studying additional loci would greatly improve these analyses. The pattern of population genetic structure in *X*. *muta* appears to be most strongly related to hydrological patterns [69] and specific life history traits. Restricted larval dispersal is a common feature in sponges (reviewed by Maldonado, 2006) [70] and might explain the observed limited recruitment of *X*. *muta* over large distances [44, 45]. Lopez-Legentil and Pawlik (2009) [46] also found significant differentiation between most distant populations of *X*. *muta* that they studied in Florida, Bahama’s and Belize, ranging in distance from 100–1000 km. Yet, the authors did not see evidence of isolation-by-distance, potentially indicating that *X*. *muta* larvae do have the ability to disperse over larger distances. However, due to the low number of I3-M11 haplotypes (n = 4) found in *X*. *muta*, the presence or absence of one specific haplotype can have a large impact on the Φst values. Using the same genetic marker in a congener, *X*. *testudinaria*, genetic divergence over small spatial scales of 2–100 km has been detected in Indonesia [54, 71]. *X*. *testudinaria* has short dispersal distances and seems to rely largely (up to 80%) on self-recruitment [72]. It has, furthermore, been proposed that *X*. *testudinaria* is a species complex with each COI haplotype possibly representing a distinct species [54, 71]. It is unclear, but possible, that this is also the case in the Caribbean *X*. *muta*.

### Population density

*M*. *cavernosa* colony densities on Saba Bank were found to be highly variable between sites (range 0.02–0.96 colonies m^-2^), but fit largely within the range of densities described by Porter *et al*. (1987) [16] for southern Florida at a depth range of 10–40 m (0.14–1.09 colonies m^-2^). However, much higher densities (up to 6.32 colonies m^-2^) have also been found throughout in the Caribbean region [15, 17]. The rather atypical flat reef character of Saba Bank, caused by continuous hydrologic and wind (including hurricanes) stress, compared to the more common massive reef structures on leeward fringing reefs around nearby islands might explain the lower densities at several sites. Also, at some sites the dominant benthic cover was sand, which likely restricted coral recruitment (*e*.*g*. SB06 with densities of 0.02 colonies m^-2^). The density of *X*. *muta* on most Saba Bank sites was comparable to previous recordings in Florida where mean densities between 0.186–0.277 m^-2^ were found at depths ranging between 15–30 m [43, 51], but data on sponge densities in the Caribbean region is scarce. There were three locations with remarkably high densities (SB05-SB07) which were characterized by substantial total sponge cover (9–13.7%) and either high algal (50%) or high sand-rubble (45%) cover [9]. The densities are similar to *X*. *testudinaria* in Indonesia. In East Kalimantan the densities ranged between 0–0.1333 in/m^2^ at depths between 5-10m [73]. In Sulawesi the densities were lower with 0.002–0.038 ind. m^-2^, yet *X*. *testudinaria* was most abundant at heavily disturbed reefs [71].

### Health status

The absence of any disease in *M*. *cavernosa* colonies confirms previous accounts [4, 8, 9] on the health status of corals on Saba Bank. This is a noteworthy comparison to other western Atlantic locations where prevalence of Black Band and White Plague Disease is much higher. Nevertheless, the *M*. *cavernosa* colonies do appear to be under stress, as exemplified by old tissue loss in the majority of the colonies and partial overgrowth of cyanobacteria, sponges or macro algae. The observed tissue loss might be the consequence of past mass bleaching events that affected reefs worldwide, including Saba Bank [9, 74]. No distinction, however, was made between different types of lesions and their relation to size or shape of the colony [75, 76]. As a result we can not be conclusive on the initial cause of the tissue. Nevertheless, *M*. *cavernosa* are known to be susceptible to bleaching, in some cases affecting up to 80% of the colonies [27, 28]. The absence of bleaching and disease on Saba Bank might partially be explained by the timing of our surveys. Although sea surface temperature late October was relatively high (up to 30°C), our study only covers a short period within the time frame (October–November 2013) in which bleaching episodes may have occurred (NOAA, website: http://www.aoml.noaa.gov/phod/cyclone/data/ca.html). To our knowledge, however, there are no reports of substantial bleaching having occurred in the Caribbean region in 2013.

In contrast, the vast majority of *X*. *muta* (> 80%) on Saba Bank showed signs of bleaching in the form of circular shaped white spots. In fact, all observed larger individuals (diameter > 50 cm) had these bleached spots (Fig 3). In addition, Sponge Orange Band Disease was found in three individuals. In comparison, the proportion of spotted bleaching in *X*. *muta* was much lower in Florida with 16–21% at 15–30 m [51]. Our observations are also considerably higher than reports by *Cowart et al*. (2006) [49] in the Florida Keys, who found cyclic bleaching in approximately 25% of the sponge population in surveys since 1997. The high proportion of bleached sponges is disconcerting given the fact that no bleached sponges were recorded on Saba Bank in 2006, during a study specifically aimed to document bleaching and disease in *X*. *muta* on the bank [5]. As bleaching in *X*. *muta* is known to be seasonal with a peak during the fall [51], the observed discrepancy might be the result of a difference in survey timing. Thacker and colleagues [5] conducted their work in January, when water temperature is generally lower compared to October (our study).

At present, the densities and genetic diversity of *X*. *muta* on Saba Bank indicate a healthy population, yet a significant portion of the sponges is affected by partial bleaching and although long term effects of this phenomenon are unknown, there is a risk of a reduction in population size. *X*. *muta* plays a crucial role in the coral reef ecosystem providing habitat complexity [77, 78] and biotope for symbionts from microbes [53, 79, 80] to invertebrates (*e*.*g*. crustaceans and brittle stars) [32, 81, 82]. Furthermore, populations of this sponge species can filter a substantial amount of water [41, 42], therewith playing an import role in nutrient fluxes and removal of particulate and dissolved organic matter *e*.*g*. [37, 83–87]. A reduction in *X*. *muta* populations would likely cause a significant change in ecosystem functioning.

## Conclusions

This is the first study to examine the potential of Saba Bank as a buffer in the region, either as a natural source of larvae to replenish genetic diversity in the region or as a storehouse of diversity that can be utilized if needed for restoration practices. Although our results are not conclusive on the direction of gene flow, they do underline the potential of Saba Bank to serve an essential ecological role within the region. There are no large land masses nearby and consequently the reefs have suffered less from pollution, coastal development and run-off. Saba Bank reefs, however, are not immune to global environmental threats such as the rise in sea surface temperature or acidification, and are subjected to local threats including fishing of natural herbivores (fish, lobsters) and explorations for natural resources as well [8]. Due to its high species and unique genetic diversity, the upstream position with respect to the wider western Atlantic, its large area of deeper reef, and relatively limited anthropogenic disturbance, Saba Bank requires further conservation efforts to serve as a potential source population to the wider Caribbean.

## Supporting Information

S1 Fig Example of Transect picture from Saba Bank site 5 (SB5). Red square (1 m^2^) overlaps with 1 m of the transect line (length between the two black dots. Only *M*. *cavernosa* (blue arrow) and *X*. *muta* (green arrow) within the red square were counted.
(EPS)

S2 Fig Marginal posterior probability distributions for isolation-with-migration (IMa2) analyses *Xestospongia muta*. Two pairwise comparisons were made: Saba Bank versus SSPC (San Salvador and Plana Cay, Bahamas, samples combined), and Saba Bank versus Stirrup Cay (Bahamas). Migration rates are given here forward in time.; line colors represent four independent simulation runs; A = simulated ancestral population.
(EPS)

S1 Table Overview of all accession numbers used in this study. including novel sequences and those obtained from GenBank.
(DOCX)

S2 Table Analysis of molecular variance (AMOVA) for both ITS (*Montastraea cavernosa*) and I3-M11 (*Xestospongia muta*) on all 11 Saba Bank sites on the south-eastern Saba Bank. (DOCX)

S3 Table Φ_ST_ values between Saba Bank sample sites for both [A] *Montastraea cavernosa* and [B] *Xestospongia muta*. Significant values (p < 0.05) provided in bold. At site SB06 no *M*. *cavernosa* were sampled.
(DOCX)

S4 Table Analysis of molecular variance (AMOVA) for both ITS (*Montastraea cavernosa*) and I3-M11 (*Xestospongia muta*) within the wider Caribbean and Gulf of Mexico. (DOCX)

S5 Table Migration analysis indices. Maximum posterior probability estimates in demographic units for four isolation-with-migration simulation runs. For details of simulations see text. N_x_ = millions of individuals in population x; A = simulated ancestral population, T = population subdivision time (millions of years ago), 2Nm_1,2_ = number of migrants population 1 (Saba Bank) receives from population 2 (SSPC or Stirrup Cay) per year. SSPC refers to data of the pooled Bahamas sites: Stirrup Cay and Plana Cay.
(DOCX)
