# Supplementary material for: Population Genetic Structure, Abundance, and Health Status of Two Dominant Benthic Species in the Saba Bank National Park, Caribbean Netherlands: Montastraea cavernosa and Xestospongia muta
Source: PLoS One. 2016 May 25;11(5):e0155969. doi: 10.1371/journal.pone.0155969 (PMC4880336; doi:10.1371/journal.pone.0155969)
Supplement: S1 Table — including novel sequences and those obtained from GenBank. (DOCX) [file pone.0155969.s003.docx]

**Supplement Table S1.** Overview of all accession numbers used in this study, including novel sequences and those obtained from GenBank.

| **ITS (*Montastraea cavernosa*)** |  | **I3-M11 (*Xestospongia muta*)** |  |
| --- | --- | --- | --- |
| Goodbody-Gringley *et al*. (2012) | HM447253 - HM447294 | Lopez-Legentil & Pawlik (2009) | EU716652 - EU716655 |
| This Study | KT254598 - HQ452962 | Montalvo & Hill (2011) | HQ452957 - HQ452962 |
|  |  | This Study | KT271771 - KT271838 |
