# Supplementary material for: Population Genetic Structure, Abundance, and Health Status of Two Dominant Benthic Species in the Saba Bank National Park, Caribbean Netherlands: Montastraea cavernosa and Xestospongia muta
Source: PLoS One. 2016 May 25;11(5):e0155969. doi: 10.1371/journal.pone.0155969 (PMC4880336; doi:10.1371/journal.pone.0155969)
Supplement: S2 Table — (DOCX) [file pone.0155969.s004.docx]

**Supplement Table S2.** Analysis of molecular variance (AMOVA) for both ITS (*Montastraea cavernosa*) and I3-M11 (*Xestospongia muta*) on all 11 Saba Bank sites on the south-eastern Saba Bank.

|  | Source of variation | df | Sum of squares | Variance component | Percentage of variation |
| --- | --- | --- | --- | --- | --- |
| ITS | Among Regions | 9 | 25.60 | 0.17036 Va | 7.15 |
|  | Within Regions | 28 | 61.98 | 2.21361 Vb | 92.85 |
|  | Total | 37 | 87.58 | 2.38396 |  |
|  | Fixation index F_ST_ | 0.07146 |  |  |  |
| I3-M11 | Among Regions | 10 | 3.982 | -0.00348 Va | 0 |
|  | Within Regions | 42 | 17.20 | 0.40952 Vb | 100 |
|  | Total | 52 | 21.13 | 0.40604 |  |
|  | Fixation index F_ST_ | -0.0857 |  |  |  |
|  |  |  |  |  |  |

*df. Degrees of Freedom*
