# Supplementary material for: Population Genetic Structure, Abundance, and Health Status of Two Dominant Benthic Species in the Saba Bank National Park, Caribbean Netherlands: Montastraea cavernosa and Xestospongia muta
Source: PLoS One. 2016 May 25;11(5):e0155969. doi: 10.1371/journal.pone.0155969 (PMC4880336; doi:10.1371/journal.pone.0155969)
Supplement: S3 Table — Significant values (p < 0.05) provided in bold. At site SB06 no M. cavernosa were sampled. (DOCX) [file pone.0155969.s005.docx]

**Supplement Table 3.** Φ_ST_ values between Saba Bank sample sites for both [A] *Montastraea cavernosa* and [B] *Xestospongia muta.* Significant values (p < 0.05) provided in bold. At site SB06 no *M. cavernosa* were sampled.

**A. *Montastraea cavernosa***

|  | SB1 | SB2 | SB3 | SB4 | SB5 | SB7 | SB8 | SB9 | SB10 | SB11 |
| --- | --- | --- | --- | --- | --- | --- | --- | --- | --- | --- |
| SB1 |  |  |  |  |  |  |  |  |  |  |
| SB2 | 0.3143 |  |  |  |  |  |  |  |  |  |
| SB3 | 0.0595 | 0.0820 |  |  |  |  |  |  |  |  |
| SB4 | 0.2393 | -0.0956 | 0.0152 |  |  |  |  |  |  |  |
| SB5 | 0.0429 | -0.1566 | -0.3846 | -0.1265 |  |  |  |  |  |  |
| SB7 | 0.2778 | -0.1020 | -0.1223 | -0.1517 | -0.2083 |  |  |  |  |  |
| SB8 | 0.1868 | -0.0093 | -0.0613 | -0.1666 | -0.1291 | -0.0926 |  |  |  |  |
| SB9 | 0.3642 | 0.3234 | -0.1875 | 0.2748 | 0.0000 | 0.0518 | 0.2499 |  |  |  |
| SB10 | 0.1938 | -0.0392 | -0.1446 | -0.0185 | -0.2126 | -0.1457 | 0.0033 | 0.0528 |  |  |
| SB11 | 0.3539 | 0.6364 | -0.3333 | 0.4286 | 0.1177 | 0.1250 | 0.2992 | -0.6923 | 0.0000 |  |

**B *Xestospongia muta***

|  | SB01 | SB02 | SB03 | SB04 | SB05 | SB06 | SB07 | SB08 | SB09 | SB10 | SB11 |
| --- | --- | --- | --- | --- | --- | --- | --- | --- | --- | --- | --- |
| SB01 |  |  |  |  |  |  |  |  |  |  |  |
| SB02 | 0.0011 |  |  |  |  |  |  |  |  |  |  |
| SB03 | -0.2027 | 0.1818 |  |  |  |  |  |  |  |  |  |
| SB04 | -0.0484 | -0.2199 | 0.1323 |  |  |  |  |  |  |  |  |
| SB05 | -0.2038 | -0.2271 | 0.0000 | -0.2992 |  |  |  |  |  |  |  |
| SB06 | -0.0613 | -0.0977 | 0.0171 | -0.1190 | -0.1739 |  |  |  |  |  |  |
| SB07 | 0.0833 | -0.0345 | 0.1892 | 0.0000 | 0.1892 | -0.0679 |  |  |  |  |  |
| SB08 | -0.0580 | 0.6842 | 0.2836 | 0.6226 | 0.3684 | 0.3425 | **1.0000** |  |  |  |  |
| SB09 | -0.1123 | -0.2358 | 0.0735 | -0.2809 | -0.3878 | -0.1451 | 0.0625 | 0.5294 |  |  |  |
| SB10 | -0.1123 | -0.2358 | 0.0735 | -0.2809 | -0.3878 | -0.1451 | 0.0625 | 0.5294 | -0.3333 |  |  |
| SB11 | -0.1300 | 0.0330 | 0.1539 | -0.0526 | -0.3333 | -0.0133 | 0.3939 | 0.1111 | -0.1667 | -0.1667 |  |
