# Supplementary material for: Population Genetic Structure, Abundance, and Health Status of Two Dominant Benthic Species in the Saba Bank National Park, Caribbean Netherlands: Montastraea cavernosa and Xestospongia muta
Source: PLoS One. 2016 May 25;11(5):e0155969. doi: 10.1371/journal.pone.0155969 (PMC4880336; doi:10.1371/journal.pone.0155969)
Supplement: S4 Table — (DOCX) [file pone.0155969.s006.docx]

**Supplement Table S4.** Analysis of molecular variance (AMOVA) for both ITS (*Montastraea cavernosa*) and I3-M11 (*Xestospongia muta*) within the wider Caribbean and Gulf of Mexico.

|  | Source of variation | df | Sum of squares | Variance component | Percentage of variation |
| --- | --- | --- | --- | --- | --- |
| ITS | Among populations | 6 | 21.11 | 0.0524 Va | 2.11 |
|  | Within populations | 128 | 325.14 | 2.5401 Vb | 97.89 |
|  | Total | 134 | 345.24 | 2.5925 |  |
|  | Fixation index F_ST_ | 0.0202 |  |  |  |
| I3-M11 | Among populations | 8 | 60.98 | 0.3545 Va | 39.04 |
|  | Within populations | 178 | 98.55 | 0.5537 Vb | 60.96 |
|  | Total | 186 | 159.53 | 0.9012 |  |
|  | Fixation index F_ST_ | **0.3904** |  |  |  |
|  |  |  |  |  |  |

df. Degrees of Freedom

Significant values (p < 0.05) given in bold.
