# Supplementary material for: Population Genetic Structure, Abundance, and Health Status of Two Dominant Benthic Species in the Saba Bank National Park, Caribbean Netherlands: Montastraea cavernosa and Xestospongia muta
Source: PLoS One. 2016 May 25;11(5):e0155969. doi: 10.1371/journal.pone.0155969 (PMC4880336; doi:10.1371/journal.pone.0155969)
Supplement: S5 Table — Maximum posterior probability estimates in demographic units for four isolation-with-migration simulation runs. For details of simulations see text. Nx = millions of individuals in population x; A = simulated ancestral population, T = population subdivision time (millions of years ago), 2Nm1,2 = number of migrants population 1 (Saba Bank) receives from population 2 (SSPC or Stirrup Cay) per year. SSPC refers to data of the pooled Bahamas sites: Stirrup Cay and Plana Cay. (DOCX) [file pone.0155969.s007.docx]

**Supplement Table 5.** Maximum posterior probability estimates in demographic units for four isolation-with-migration simulation runs. For details of simulations see text. N_x_ = millions of individuals in population x; A = simulated ancestral population, T = population subdivision time (millions of years ago), 2Nm_1,2_ = number of migrants population 1 (Saba Bank) receives from population 2 (SSPC or Stirrup Cay) per year. SSPC refers to data of the pooled Bahamas sites: Stirrup Cay and Plana Cay.

|  | Saba Bank (1) vs. SSPC (2) | | | | | |  | Saba Bank (1) vs. Stirrup Cay (2) | | | | | |
| --- | --- | --- | --- | --- | --- | --- | --- | --- | --- | --- | --- | --- | --- |
| run | *N_1_* | *N_2_* | *N_A_* | *T* | *2Nm_1,2_* | *2Nm_2,1_* |  | *N_1_* | *N_2_* | *N_A_* | *T* | *2Nm_1,2_* | *2Nm_2,1_* |
| 1 | 0.91 | 0.65 | 0.035 | 4.6 | 0.35 | 1.45 |  | 1.5 | 0.77 | 8.2 | 1.7 | 0.77 | 0.023 |
| 2 | 0.81 | 0.67 | 0.30 | 9.4 | 0.25 | 1.35 |  | 1.5 | 0.74 | 8.3 | 1.8 | 0.72 | 0.025 |
| 3 | 0.96 | 0.63 | 0.012 | 5.9 | 0.55 | 1.35 |  | 1.5 | 0.77 | 8.4 | 2.0 | 0.77 | 0.025 |
| 4 | 0.71 | 0.70 | 3.6 | 0.024 | 0.45 | 0.84 |  | 1.6 | 0.74 | 8.1 | 1.7 | 0.72 | 0.025 |
